# A Comparison of Risks and Benefits Regarding Hip Arthroplasty Fixation

**DOI:** 10.5435/JAAOSGlobal-D-21-00014

**Published:** 2021-11-01

**Authors:** Julia Matthias, Mathias P. Bostrom, Joseph M. Lane

**Affiliations:** From the Metabolic Bone Disease, Department of Orthopaedic Surgery, Hospital for Special Surgery (Dr. Matthias and Dr. Lane), and the Division of Adult Reconstruction and Joint Replacement, Department of Orthopaedic Surgery, Hospital for Special Surgery, New York, NY (Dr. Bostrom).

## Abstract

Since the field-changing invention of noncemented hip arthroplasty fixation in the 1980s, noncemented fixation has been progressively replacing cemented fixation. However, analyses of fixation frequencies reveal new patterns in cement versus noncemented preferences. Although cementation is again gaining ground in the United States, noncemented models remain the dominant fixation mode, seen in more than 90% of all hip arthroplasties. This stark preference is likely driven by concerns regarding implant durability and patient safety. Although advances in surgical techniques, intensive perioperative care, and improved instrument have evolved in both methods, data from large arthroplasty registries reveal shifting risks in contemporary hip arthroplasty, calling the use of noncemented fixation into question. Varying risk profiles regarding sex, age, or health comorbidities and morphological and functional differences necessitate personalized risk assessments. Furthermore, certain patient populations, based on the literature and data from large registries, have superior outcomes from cemented hip arthroplasty techniques. Therefore, we wanted to critically evaluate the method of arthroplasty fixation in primary hip arthroplasties for unique patient populations.

Hip arthroplasty is one of the greatest achievements of modern orthopedic surgery.^1^ Although already one of the most common orthopedic surgeries, it is done with steadily increasing frequency because of an aging global population.^2^ Total hip arthroplasty (THA) is the leading elective surgical therapy for primary and secondary osteoarthritis. It results in sustainably decreased mortality for 10 years postoperatively compared with a matched, nonoperated control population.^3^ Hemiarthroplasties (HAs) are commonly done to treat femoral neck fractures (FNFs), to which the elderly population is particularly vulnerable.^4^ In the setting of FNF, HA permits rapid resumption of premorbid levels of activity. Hip arthroplasty is thus considered one of the most successful orthopedic surgical procedures.

In the 1980s, advances in fixation methods eliminated the need for cement and revolutionized hip arthroplasty.^5^ Noncemented prostheses could be tightly inserted within the medullary canal using press-fit fixation, without the use of cement. Moreover, these procedures were generally shorter,^6,7^ with attendant reductions in postoperative infection risk.^8,9^ More recent data, however, suggest that surgery time and blood loss have equalized between both fixation methods,^10^ likely reflecting surgical and prosthetic advances. In particular, the use of noncemented cup fixation supplanted cemented cups because of outstanding functional outcomes and is becoming the new benchmark.

After their introduction, noncemented hip arthroplasties have progressively replaced cemented alternatives (Figure 1). In the United States, up to 93% of hip arthroplasties for osteoarthritis use noncemented fixation,^11^ likely motivated by risk of cement-induced cardiovascular events and adverse long-term outcomes related to early bone-cement interface loosening.^12^ Aggressive marketing and rapid introduction of improved noncemented designs were also influential in the adoption of noncemented arthroplasty as a preferred technology. Interestingly, arthroplasty fixation has undergone a new change of direction within the past 10 years. The 2020 AJRR Annual Report revealed a nadir in the frequency of cemented arthroplasty in 2013, used only in 2.4% of all primary THAs in the United States.^13^ Since then, cementation has steadily increased to 5.3% of all arthroplasties in 2019. Still, noncemented THAs seem to be the preferred method in the United States.^13^ In addition, the national joint registry of England and Wales observed a gradual shift in fixation choice in favor of hybrid procedures versus purely noncemented alternatives.^14^

**Figure 1:**
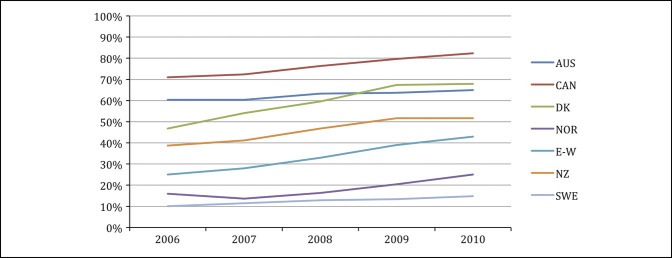
Graph showing the percentages of noncemented THAs of all primary THAs per year from 2006 to 2010. Reprinted from A review of current fixation use and registry outcomes in total hip arthroplasty: the noncemented paradox, Troelsen A. et al, 2013, by *Clinical orthopaedics and Related Research*. AUS = Australia, CAN = Canada, DK = Denmark, E-W = England-Wales, NOR = Norway, NZ = New Zealand, SWE = Sweden, THA = total hip arthroplasty

An evidence-based study of arthroplasty fixation methods is required to determine the optimal fixation method for various patient groups. Unfortunately, much of the scientific literature on this topic relies on outdated databases populated with outmoded surgical techniques and implants. This article aims to provide an updated review of the most recent literature comparing risks and benefits of both fixation techniques in distinct patient populations. We focused on the most frequent serious complications of primary hip arthroplasties, such as periprosthetic fractures, prosthetic joint infections, and aseptic loosening, and we compared mortality and functional results of each fixation method. In doing so, we aimed to critically evaluate what is the best method of arthroplasty fixation for unique patient populations.

## An Overview of Threats to Prosthetic Longevity

### Periprosthetic Fractures

A periprosthetic fracture (PPF) is a severe complication of fixation, with serious patient morbidity. Noncemented prostheses pose an increased risk^15^ for the development of PPF compared with its cemented counterparts. Because the stability of the femoral stem requires a tight fit with the medullary canal, greater force is needed to insert the noncemented stem. A robust baseline bone stock reduces the risk of perioperative trauma-induced PPF. However, most patients receiving hip arthroplasty are elderly population and experience diminished bone quality.

### Periprosthetic Joint Infection

Periprosthetic joint infections (PJIs) are a significant cause of THA failure resulting in significant morbidity. Biofilm formation on the implant surfaces promotes microorganism growth, often requiring complex interdisciplinary treatment. Importantly, the mode of fixation affects the risk of PJI. Heat generation during the cementation process can induce bone necrosis, creating favorable microbial growth conditions. However, in practice, antibiotic-infused cement enables local, targeted infection control because antibiotics are released directly into the adjacent tissues. Far greater local antibiotic concentrations can be achieved compared with systemic administration, simultaneously limiting toxicity associated with systemic administration.^16,17^

### Aseptic Loosening and Functional Quality

Aseptic loosening is defined as the loss of fixation without associated infection, a process that can be initiated immediately after joint replacement because of insufficient intraoperative fixation but can also appear years later through osteolytic processes around the implant. Aseptic loosening is particularly serious because it cannot be treated nonoperatively. In the early days of THA, aseptic loosening was a dreaded yet relatively common long-term complication. Periprosthetic polyethylene and metal debris from prosthetic wear were a major cause of this complication.^18,19^ However, because aseptic loosening was initially believed to be secondary to toxic effects of cement on bone tissue, it was colloquially referred to as cement disease.^12^ Noncemented fixation was, therefore, believed to be a solution to cement disease; however, it was soon recognized that noncemented implants could instigate early prosthetic loosening because of insufficient initial prosthetic integration or osteolysis.

### Mortality and Bone Cement Implantation Syndrome

Elective THA is associated with very low short-term mortality and is even associated with sustained reduced long-term mortality.^3,20^ Still, initial observations of perioperative cardiovascular incidents after the introduction of the cementation procedure^21,22,23,24^ gave rise to the term bone cement implantation syndrome. Hypothetically, these potentially life-threatening postoperative cardiovascular events resulted from fat and bone marrow emboli to the pulmonary arterial tree, caused by surgical manipulation and cement-generated pressure within the medullary cavity.^25^ However, surgical procedures and hip prostheses are constantly improving, as are the capabilities of anesthesia and intensive perioperative care, cumulatively leading to an overall drop of perioperative mortality during the past decades.^26^ More recent studies comparing the perioperative mortality of cemented and noncemented implants demonstrated similar morbidity and mortality between cemented and noncemented arthroplasty.

### Cemented and Noncemented Fixation in Primary Elective Total Hip Arthroplasties

The elective implantation of a THA requires careful consideration of various patient-specific factors, guiding both the choice of implant and fixation method.

**Figure 2:**
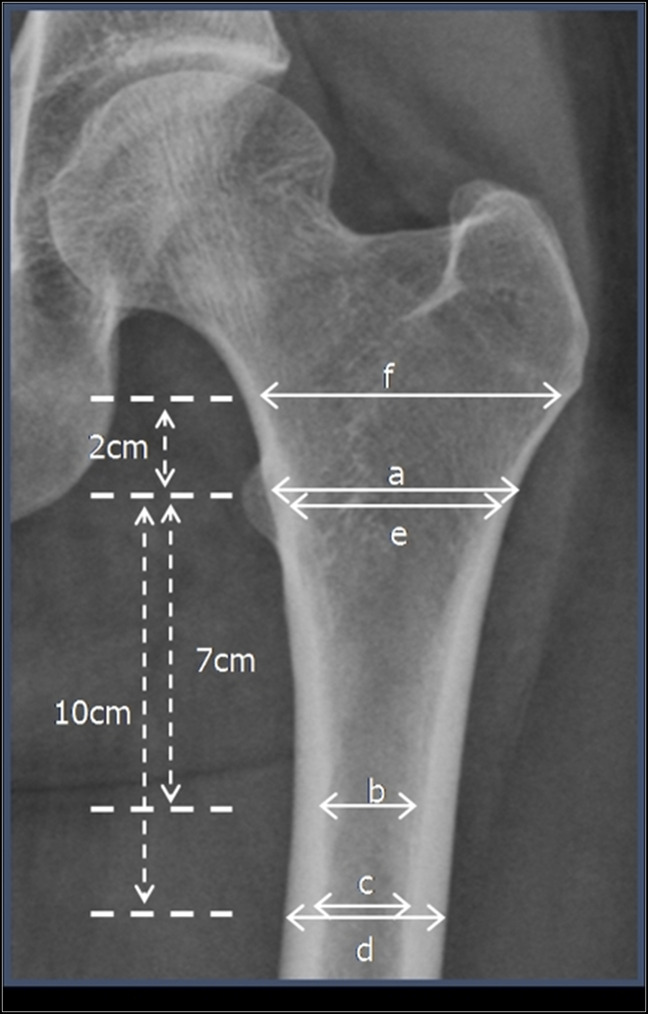
Radiograph showing analysis to ascertain the proximal femoral morphology. Reprinted from Early Post-operative Periprosthetic Femur Fracture in the Presence of a Non-cemented Tapered Wedge Femoral Stem, Cooper HJ et al, 2010, by the *HSS Journal: the Musculoskeletal Journal of Hospital for Special Surgery*. cortical index = a/b, canal cone ratio = c/d, canal to calcar ratio = c/e, canal-flare index = f/c

One such factor is the geometry of the proximal femur. The canal to calcar ratio (Figure 2) describes the shape of the proximal femur and was subdivided by Lawrence Dorr into three classes (A, B, and C) with increasing quotients: a small quotient describes a conically shaped proximal femoral canal and a high quotient indicates a wide femoral canal with a resultant cylindrical shape.^27^ Thus, a wide femoral isthmus requires a larger stem to assure a snug fit of the femoral stem. However, because the femoral device is tapered in shape, a large noncemented stem cannot be placed into a Dorr C femur without harming the calcar or neck bone stock, resulting in greater than 5x increase in PPF after noncemented implants compared with Dorr B femur.^28^ Dorr et al, therefore, recommended cemented implants in patients with Dorr C femur.^27^ Interestingly, patients with Dorr A femur with sharp narrowing of the proximal femoral canal experience an increased rate of PPF and loosening after noncemented implants,^29^ which, similar to Dorr C, is secondary to mismatch between a stem and femur.

Bone stability and quality is another factor that naturally comes to mind when considering prosthesis longevity and complication rate. Osteoporosis is a frequent cause of diminished bone quality, especially in the elderly population, which can be evaluated radiographically using the canal bone ration (CBR, Figure 2).^30^ To determine how a deficient bone stock correlates with implant survival, Yang et al^31^ studied cemented versus noncemented implant survival in osteoporotic patients. They found a high implant failure rate of 14.8% versus 7.6% in noncemented versus cemented implants, respectively. Moreover, bone stock quality is a risk factor for periprosthetic fracture: Kouyoumdjian et al^32^ found a strong correlation between a high CBR and the incidence of PPFs in patients older than 75 years receiving noncemented HA after FNF, favoring cementation when the CBR exceeds 0.49.

Women represent another patient group prone to bone quality impairment. Because postmenopausal women experience diminished bone quality earlier than men of the same age, sex may play a role in the fixation mode decision process. Indeed, Dale et al ^33^ found that women receiving noncemented femoral components averaged a 19-fold increased risk of PPF-associated revision compared with those receiving cemented implants. Even after 10 years of follow-up, the noncemented group of women still had significantly increased risk of fracture as opposed to men.

Remarkably, patients receiving cemented arthroplasties are approximately 10 years older than those receiving noncemented arthroplasties,^34,35^ making a comparison of overall mortality between the arthroplasties difficult. However, analysis by Ekman et al^35^ of the arthroplasty register in Finland, where noncemented THAs continue to be commonly done in elderly and frail patients, revealed similar perioperative adjusted mortality for both THA techniques. Instead, increased long-term mortality associated with cemented THA seems to be related to comorbid conditions that are more frequent in the elderly population. However, a slight but nonsignificant increase in mortality was seen in the most fragile patient group.^36^ Dale et al ^34^ found that mortality was comparable with both fixation types when selecting for a subgroup of commonly used contemporary and well-documented THA methods. However, Garland et al ^37^ found an increase in mortality within the first 14 days of surgery in the cemented THA group, compared with matched untreated control subject in the general population. In this study, patients receiving hybrid THAs consisting of a cemented stem and noncemented acetabular cup showed an increased perioperative mortality compared with patients receiving a reverse hybrid THA (a noncemented stem with a cemented acetabular cup). This analysis, which selected comparable, relatively young and healthy patients, suggested that cementation of the femur is associated with a very small but statistically significant increase in perioperative mortality, accounting for five additional deaths per 10,000 patients.

Another complication that seems to be closely related to aging and bone quality is aseptic loosening. Tanzer et al,^38^ comparing contemporary cemented and noncemented designs in patients older than 74 years, found fewer cases of aseptic loosening in cemented implants for both osteoarthritic and FNF patients. Dale et al,^33^ on the other hand, reported a markedly decreased incidence of noncemented stem aseptic loosening over the long term (measured at 10 years postsurgery). However, this study did not differentiate between early and late implant failure, so the possibility of a higher rate of early postoperative loosening in noncemented stems cannot be excluded. To examine the exact relationship between bone quality and aseptic loosening, Yang et al^31^ analyzed early implant failure due to aseptic loosening in osteoporotic patients. In patients aged 60 to 80 years, they found an early failure rate of 26.6% in patients with noncemented implants compared with 16.8% in those with cemented implants. Patients with noncemented implants had a markedly inferior Harris hip score (HHS; evaluates pain, function, deformity, and range of hip motion) at 3 months postsurgery. Interestingly, a trend of increased discomfort was found in both resting and active states in these patients.^31^ Goyal et al^39^ also found better short-term clinical outcomes after cementation, with superior improvement in pain control and earlier full weight-bearing tolerance. In line with other studies reporting elevated risk of early noncemented implant failure in osteoporotic patients, Aro et al^40^ found positive correlations among low bone mineral density, prosthetic subsidence, and delayed translational stability of the femoral stem. Furthermore, they found increasing age a risk factor of delayed rotational stability.^40^ As early subsidence within the first 24 months serves as an independent risk factor for aseptic loosening,^41^ cemented fixation is recommended in elderly osteoporotic patients.

Another postoperative complication that patients with many comorbidities are particularly prone to are PJIs. Published data confirm the value of local antibiotic application for deep joint infections. Zhang et al^42^ observed a reduced rate of deep PJI on use of combined systemic and local antibiotics through antibiotic-infused bone cement (AIBC) versus systemic treatment alone. AIBC decreases the risk of revision for PJI by one-third compared with standard cement.^43^ Superficial infection prevention, however, required systemic antibiotics because patients who were solely treated with AIBC exhibited an increased risk of superficial skin infections.^42^ This might be due to a high concentration gradient, with sufficient local antibiotic concentrations at the hip joint while concentrations steadily decrease toward the superficial surgical site, dropping below minimal inhibitory concentration. Systemic antibiotics are needed to address superficial surgical site infections. Interestingly, Zhang et al^42^ detected in their meta-analysis that AIBC did not show protective effects for deep joint infections when laminar flow was present during the surgical procedure. This might be related to the superior preventive effect of laminar flow, reducing the risk of deep joint infection to such low levels that AIBC did not demonstrate further risk reduction. The use of AIBC might, therefore, be of particular importance when laminar flow is not available. When comparing AIBC with noncemented prosthetic placement, Colas et al^44^ found a significantly reduced rate of revision for any reason after AIBC arthroplasty compared with noncemented placement (2.4% versus 3.3%), whereas Kunutsor et al,^9^ who specifically compared the PJI incidence, found a comparable PJI risk in their meta-analysis. Thus, preventive measure against PJIs in vulnerable patients by using AIBC might be beneficial.

Perioperative and postoperative complications such as PPFs, aseptic loosening and PJI lead to revision surgery and are associated with morbidity and mortality.^45,46^ This is particularly important to consider in patients who are elderly and already present with significant morbidity due to preexisting diseases. Higher ASA scores, for instance, have recently been implicated as an independent risk factor of PPF,^32,33,47,48^ rendering this population vulnerable to intraoperative trauma and poorer outcomes.^49,50^ A comparative outcome study of modern arthroplasty designs found a higher incidence of PPF in noncemented prostheses within the first 3 postoperative months in an elderly patient population (older than 74 years).^38^ In this group, twice as many revision surgeries were required when compared with patients receiving cemented arthroplasties. Although patients younger than 55 years receiving noncemented implants may have a slightly increased short-term revision rate,^51^ their superior general health may portend better long-term outcomes. Importantly, although cementation has a reduced immediate risk of PPF, its removal during revision surgery is accompanied by a high fracture rate.^52^ PFFs can be categorized by the Vancouver classification regarding the location of the fracture, thereby guiding treatment.^53^ Although fractures around stable implants can be treated conservatively or with open reduction and internal fixation,^54^ unstable implants require revision arthroplasty,^54^ which, again, is associated with high morbidity and mortality.^45,46^ Extensively porous-coated or titanium modular fluted tapered revision stems can be used as noncemented alternatives^55–57^ to restore long-term implant stability. However, Munegato et al^55^ detected a dislocation rate as high as 16% in patients receiving a titanium modular fluted tapered stem. Further complicating the matter, cement can extravasate into the fracture gap during PFF revision, potentially causing late-onset periprosthetic fractures and malunion, whereas porous-coated stems are associated with a low refracture rate of 3.4%^56^ and a stable bony ingrowth of 98% or above.^56,57^ The risk of cortical perforation with extrusion of cement increases even further during the process of previous cement removal of primarily cemented THAs. Briant-Evans et al,^58^ therefore, suggested a cement-in-cement approach in selected cases with a well-preserved preexisting cement mantle. This suggestion coincides with a recent systematic review by Xará-Leitner et al, confirming a very low rate of intraoperative complications such as PFF (5.3%) during cement-in-cement approach revision.^59^

Overall, the data suggest that elderly patients with reduced bone quality may benefit from the immediate postoperative stability and decreased bone trauma offered by cemented implants. Interestingly, time trend analyses of large hip arthroplasty registries have shown reduced aseptic loosening revision for more recent noncemented implants,^51,60^ likely reflecting improved designs and surgical advances in recent years.

### Cemented and Noncemented Arthroplasties on Femoral Neck Fractures

FNFs are associated with frailty, morbidity, and poor bone quality^61^—all previously established as threats to the longevity of noncemented arthroplasties in the previous section. In such populations, the use of noncemented implants should, therefore, be approached with caution, given their 5x increased risk of requiring revision surgery due to PPFs.^62,63^

In patients with FNF, noncemented THA fixation has also been associated with double the rate of aseptic loosening, significantly lower HHS, and more pain, requiring surgical revision.^64^ Similarly, a recent randomized controlled trial by Clement et al^10^ comparing cemented versus noncemented THA after FNF revealed a clearly increased intraoperative complication rate for noncemented implants, leading to an early termination of the study. The poor bone quality within the FNF study population was suspected as the cause for this discrepancy. Kristensen et al found a 3.9-fold increased risk for aseptic loosening in noncemented implants after FNF. Although no differences in quality of life or functional scores between the fixation modes were seen, Inngul et al^65^ found an advantage of cement at 1 year postsurgery, with higher HHS and quality of life scores (EQ-5D) and higher musculoskeletal functional scores (SMFA; assesses daily activities, emotional status, mobility, and subjective restrictions due to pain).

**Figure 3:**
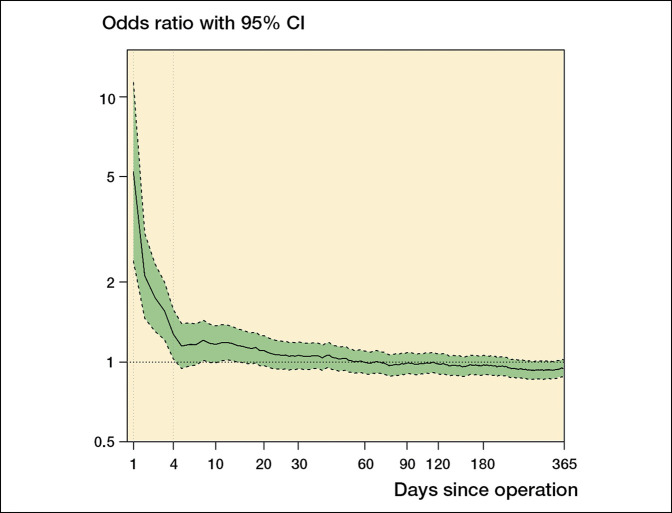
Graph showing the relative and cumulative risk of death in patients after femoral neck fracture receiving cemented hemiarthroplasty compared with patients receiving noncemented hemiarthroplasty. (Reprinted from Yli-Kyyny T, Sund R, Heinanen M, Venesmaa P, Kroger H: Cemented or uncemented hemiarthroplasty for the treatment of femoral neck fractures? *Acta Orthop* 2014;85:49-53.) The mortality is significantly higher after cemented fixation until day 4.

Arthroplasties as a treatment of FNF are associated with greater mortality than elective THAs. This association seems to be greater particularly with cemented arthroplasties (Figure 3). Yli-Kyyny et al^66^ found increased early mortality (until postoperative day four) and incidence of fat embolism after cementation. Although not statistically significant, early perioperative mortality trended down postoperatively, eventually falling below the mortality of noncemented implant recipients. From day 5 onward, the difference in mortality between these groups were indistinguishable to one-year postoperatively. This was even true when only considering high throughput orthopedic centers, thus minimizing skewed data points from inexperienced surgeons. Moreover, other studies confirmed no notable difference in mortality for FNF arthroplasty, regardless of cementation status, up to 9 years postoperatively. ^36,62,63,67,68,69,70^ Notably, despite a potential increase in early perioperative mortality among patients receiving cemented implants for treatment of FNF, this patient population seems to benefit from lower morbidity overall. For instance, Duijnisveld et al^70^ noted a significantly higher revision rate after noncemented arthroplasties in FNF patients nine years postoperatively and even as early as one year postoperatively, Yli-Kyyny et al^66^ reported more mechanical complications and revision surgeries in patients receiving noncementedarthroplasties.

Although an increase in perioperative mortality is reported after femoral stem cementation, this risk is more pronounced in frail and comorbid patients. Short-term and long-term advantages of cemented hip replacement after FNF, such as decreased PFF, PJI, and improved early postoperative function (allowing for early weight-bearing), may outweigh the slight increase in perioperative mortality.

## Summary

Since the introduction of noncemented implants in hip arthroplasty, a global shift toward the use of noncemented prostheses is observed. Despite the recent rise in the use of cemented prostheses, noncemented hip arthroplasty still significantly dominates the use of cemented fixation in the United States. Advantages and disadvantages of cementation continue to be hotly debated in the literature.

For early postoperative fixation, noncemented arthroplasty relies on press-fit fixation achieved through forceful impaction, whereas long-term fixation requires a bone-prosthesis interlock mediated by bone ingrowth into the prosthetic surface. Therefore, both early and long-term fixation require healthy, regenerative bone stock. However, most patients receiving hip arthroplasty are comparatively old, report one or more comorbid conditions, and likely possess suboptimal bone stock. Therefore, most THA patients are susceptible to complications associated with noncemented fixation, including PPF, PJI, and aseptic loosening. The immediate stability provided by cementation provides functional advantages in the early postoperative period and allows for rapid, pain-free full weight-bearing, and recovery, which may greatly benefit in this frail patient population. Osteoporosis or clinical signs of reduced bone stock (such as low-energy fractures or thin bone cortices on imaging) should, therefore, influence surgical decision making toward immediate stability of the cemented arthroplasty stems. Radiographic geometry of the proximal femur should also be considered because Dorr C femurs are likely to be incongruent to a tapering noncemented implant, exposing the patient to an increased risk of periprosthetic fractures.

Intraoperative life-threatening complications induced by cement, such as bone cement implantations syndrome, may call for noncemented arthroplasty in patients with multimorbidity. However, this increased perioperative and postoperative mortality from cemented hip arthroplasty does not remain elevated beyond the early postoperative stage. Advances in orthopedic surgical technique and in anesthesiologic intensive care have markedly reduced perioperative mortality, operation time, and need for transfusion. The remaining perioperative risk in cemented arthroplasty patients with multiple comorbidities may be outweighed by early postoperative advantages of cemented implants, and such advantages should be determinative in selecting cemented fixation. Cementation may, therefore, be a viable alternative in elderly patients and those with reduced bone quality and unfortunate geometry.

Young patients with healthy bone stock generally benefit from noncemented fixation. In this patient group, noncemented implants are associated with lower risk of late aseptic loosening and longer implant survival. Although late aseptic loosening secondary to cementation is a real complication and, thus, of particular concern in young patients with longer life expectancies, time trend analyses reveal a decreasing incidence of aseptic loosening in recent, contemporary hip cemented arthroplasties, likely related to prosthetic and surgical advances. Because of better bone health and stability, incidence of periprosthetic fracture and early aseptic loosening after noncemented arthroplasty are lower in this group. Delayed full weight-bearing from early complications may be more easily managed because of better overall health in the younger patient population. Furthermore, bone density is better preserved around certain noncemented implants compared with cemented stems,^71^ thereby offering the possibility of better bone quality and stability surrounding the implant in young patients. Unfortunately, because of longer life expectancy and prosthetic demands, revision risk in young patients remains comparatively high.

Despite the current predominance of noncemented fixation, certain patient populations have superior outcomes with cemented hip arthroplasty compared with noncemented fixation. Individual patient characteristics should be considered thoroughly when deciding which hip arthroplasty fixation mode is best suited for a given patient.
